# TouchScreen-based phenotyping: altered stimulus/reward association and lower perseveration to gain a reward in mu opioid receptor knockout mice

**DOI:** 10.1038/s41598-019-40622-6

**Published:** 2019-03-11

**Authors:** Laura-Joy Boulos, Md. Taufiq Nasseef, Michael McNicholas, Anna Mechling, Laura Adela Harsan, Emmanuel Darcq, Sami Ben Hamida, Brigitte Lina Kieffer

**Affiliations:** 1 Douglas Mental Health Institute, Department of Psychiatry, McGill University, Montreal, Quebec Canada; 2 Department of Radiology, Medical Physics, Medical Center University of Freiburg, Faculty of Medicine, University of Freiburg, Freiburg, Germany; 3 Engineering science, computer science and imaging laboratory (ICube), Integrative Multimodal Imaging in Healthcare, University of Strasbourg – CNRS, Strasbourg, France; 4 Department of Biophysics and Nuclear Medicine, Faculty of Medicine, University Hospital Strasbourg, Strasbourg, France

## Abstract

While the contribution of Mu Opioid Receptors (MORs) to hedonic aspects of reward processing is well-established, the notion that these receptors may also regulate motivation to gain a reward, and possibly other related cognitive dimensions, has been less investigated. The prefrontal cortex (PFC) is a critical site for these processes. Our previous functional magnetic resonance imaging study found alterations of functional connectivity (FC) in reward/aversion networks in MOR knockout mice. Here we pursued voxelwise seed-based FC analyses using the same dataset with a focus on the PFC. We observed significant reduction of PFC FC in mutant mice, predominantly with the nucleus accumbens, supporting the notion of altered reward-driven top-down controls. We tested motivation for palatable food in a classical operant self-administration paradigm, and found delayed performance for mutant mice. We then evaluated motivational and cognitive abilities of MOR knockout mice in TouchScreen-based behavioral tests. Learning was delayed and stimulus/reward association was impaired, suggesting lower hedonic reward value and reduced motivation. Perseverative responses were decreased, while discriminatory behavior and attention were unchanged, indicative of increased inhibitory controls with otherwise intact cognitive performance. Together, our data suggest that MORs contribute to enhance reward-seeking and facilitate perseverative behaviors. The possibility that MOR blockade could reduce maladaptive compulsivity deserves further investigation in addiction and self-control disorder research.

## Introduction

The mu opioid receptor (MOR) reduces pain and produces euphoria, and these major aspects of MOR function are well-established^1,2^. Reinforcing effects of both opioid and non-opioid drugs of abuse, as well as maternal attachment^3^ are reduced in MOR knockout mice, demonstrating the central role of MOR in mediating hedonic properties of both artificial and natural rewards^4,5^. Further, our recent analysis of whole brain functional connectivity (FC) in mice lacking the MOR gene using Resting-state functional Magnetic Resonance Imaging (Rs-fMRI) revealed major alterations in the MOR-enriched reward/aversion networks^6^, supporting the predominant MOR function in maintaining hedonic homeostasis.

Beyond hedonic processing, the question of whether MOR is involved in other reward-associated processes, such as motivation to gain a reward and decision-making, has been less studied. In an earlier report, MOR knockout mice showed lower levels of self-administration in FR3 and progressive ratio schedules^7^, for both regular chow and sucrose, suggesting a significant MOR implication in the motivation to eat^8^. Another study used Pavlovian Instrumental Transfer to assess motivational processes in MOR and delta opioid receptor (DOR) knockout mice^9^. MOR mutant mice showed normal transfer but reduced sensitivity to outcome devaluation, whereas the opposite was observed for DOR knockout mice, and local pharmacology using specific antagonists identified the nucleus accumbens (NAc) shell and core as main sites mediating these aspects of opioid-regulated incentive motivation^9^. These experiments provide evidence that opioid receptors, and MORs in particular, do more than just attributing hedonic values to rewards, and contribute to reward-guided decision making^10^.

**Figure 1:**
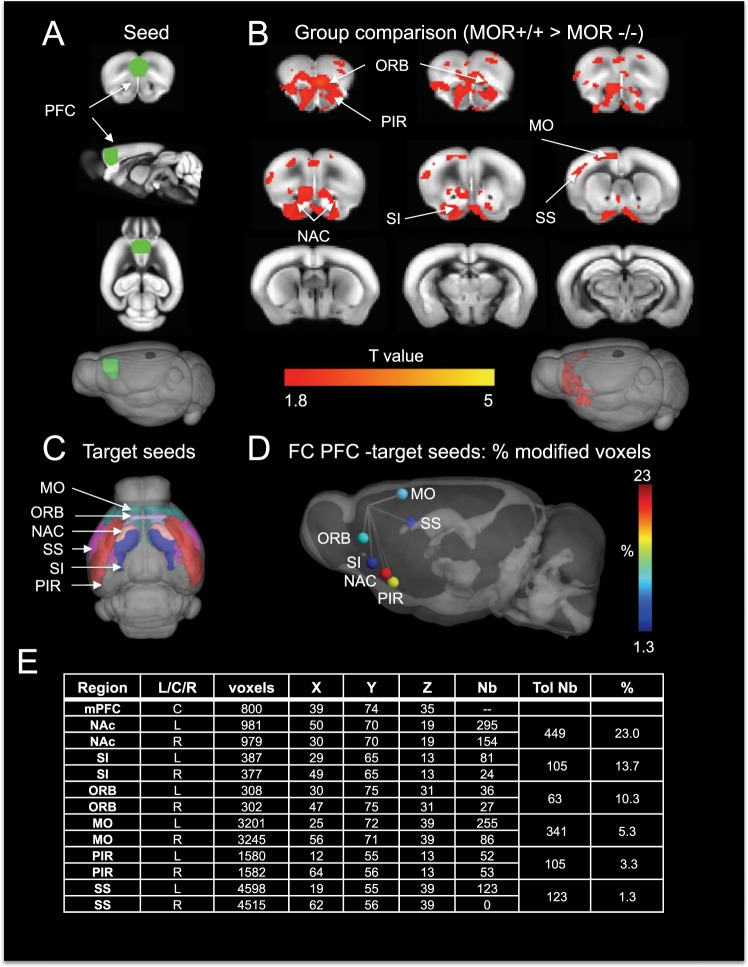
Seed-based analysis of Rs-fMRI data from MOR knockout mice reveals lower PFC connectivity with several brain regions, with a major effect for PFC-NAc. (**A**) Localization of the PFC seed (green) in 2D (3 top panels) and 3D (bottom panel) overlaid on the Allen Mouse Brain Connectivity Atlas. (**B**) Group comparison T values by one tail t-test (T > 1.8, cc = 0.05, n = 13/group, FWER corrected) shows voxels with higher functional connectivity in MOR+/+ compared to MOR−/− groups. The color bar indicates the corresponding significant T-value scaling. (**C**) Localization of the six identified target seeds for quantitative analysis in 3D representation overlaid on the Allen Mouse Brain Connectivity Atlas. (**D**) Schematic representation of the six seed regions, color-scaled to indicate the percent voxels showing significant changes (from Table in E). (**E**) Table showing regions with highest to lowest FC modifications with the PFC seed. Columns from left to right show: the region name (Region), laterality (Left, L; Centre, C; Right, R), the total number of voxels per region (voxels), coordinates of the region (X,Y,Z), the number of voxels showing significant changes across genotypes (Nb) and the resulting total number per region (Tol Nb), and the percent voxels (%) with significant changes across genotypes within the region. MO, somatomotor areas; NAc, nucleus accumbens; ORB, orbitofrontal cortex; PIR, piriform area; SI, substantia innominate; SS, somatosensory cortex. Brain section figures are modified from Allen Mouse Brain Connectivity Atlas API. Available from: http://mouse.brain-map.org/experiment/thumbnails/100048576?image_type=atlas.

Goal-directed behaviors engage the striatum (including the NAc) and notably also the prefrontal cortex (PFC), and these two brain structures are major mesocorticolimbic centers integrating bottom-up reward information and top-down executive controls to guide decision-making^11^. Our recent Rs-fMRI study, comparing FC patterns in MOR knockout mice and their controls using a data-driven approach, allowed establishing a signature of MOR activity on whole-brain connectivity^6^. Here we designed a hypothesis-driven seed analysis of the same datasets to specifically interrogate PFC connectivity patterns in MOR mutant mice. We found significant weakening of PFC connectivity with several brain areas, and highest alterations were found with voxels overlapping the NAc (Fig. 1). This observation supports the notion that MORs contribute to reward-driven motivation and action, as well as perhaps other aspects of executive controls involving the PFC^12,13^.

To further test this hypothesis, we designed a behavioral testing series using the increasingly used TouchScreen-based approach to address reward-driven behaviors in MOR knockout mice. This experimental setting differs from traditional operant self-administration testing, as the approach examines broader aspects of decision-making, including cognitive controls. The TouchScreen protocol also involves operant responding to highly palatable food, and our experimental design included (i) an autoshaping protocol that provides measures of associative learning and reward value, and (ii) a 5-Choice Serial Reaction Time task (5-CSRTt) providing measures of attention, motivation and inhibitory controls (including motor impulsivity and compulsivity)^14^, which we previously found altered in MOR knockout mice using a signaled nose-poke task^15^. Collectively, data from this study substantiate previous evidence that, beyond a major role in hedonic processing, MOR activity favors motivation to gain a reward, and may also facilitate compulsive-like behaviors.

## Material and Methods

### Animals

MOR−/− mice lacking MOR were produced as previously described^16^. All experiments were performed following the guidelines on animal experimentation established by the Canadian Council of Animal Care and animal protocol was approved by the Animal Care Committees of McGill University/Douglas Mental Health University Institute, Montreal, Canada (#2014-2018/7466). See Supplemental information for detailed description.

### Resting state fMRI data analysis

The acquisition of Resting-state fMRI images is described in our previous study^6^, and raw data from these acquisitions were used for the present analysis. The preprocessing pipeline involved the following steps: (i) denoising using Advanced Normalized tools (ANTs) (https://github.com/ANTsX/ANTs/blob/master/Examples/DenoiseImage.cxx); (ii) motion correction using ANTs and FSL (https://fsl.fmrib.ox.ac.uk/fsl/fslwiki); (iii) registration following two steps, first normalizing all the subject’s data using a template created by the existing subject’s mean volumes using ANTs, and second registering all these normalized data with the high-resolution Allen brain template^6^ via MAGeT tool^17^; (iv) smoothing with a Gaussian Kernel of full width half maximum (FWHM) at 0.3 by AFNI (https://afni.nimh.nih.gov/); (v) cropping using a brain mask generated according to gray and white matter segmentation using Allen template space and applied to all subjects; (vi) bandpass filtering at a frequency of window between 0.01 Hz to 0.1 Hz by AFNI tool.

Seed-based voxelwise analysis was then used to study functional connectivity patterns of the PFC, as described in our previous work^18^. In brief, PFC 3D bilateral seeds were constructed manually following the guidance and location to known neuroanatomical structures from the 2011 Allen Institute for Brain Science. Allen Mouse Brain Connectivity Atlas API. Available from: http://mouse.brain-map.org/experiment/thumbnails/100048576?image_type=atlas. Correlation analysis was done as follows: (i) the mean of PFC seed time series was used to produce a whole brain correlation map, (ii) transformed into Fishers Z scores and (iii) and tested for significant differences between MOR knockout and control groups using t-test (threshold T > 1.8; significance/alpha level 0.05, one tail). All results were family-wise error rate (EWER) corrected following clustering with significance/alpha level 0.05^19^. For 3D representation of the mouse brain for all the data, we used Mango (http://ric.uthscsa.edu/mango/) and BrainNet Viewer (https://www.nitrc.org/projects/bnv/) tools.

#### Operant Self-Administration

See Supplemental information for detailed description.

### Touchscreen Experiments

The different paradigms using the TouchScreen are schematized in Supplementary Fig. S1. During all Touchscreen experiments, mice underwent a restriction diet (Hab 1), to maintain all animals at approximately 85% of respective baseline free feeding weight. Mice underwent testing for one session per day, six days per week. Experimental initiation times for individual mice were kept consistent throughout the study, with four mice of a given genotype being tested at one time. Testing times were counterbalanced across experimental groups.

### Autoshaping

#### Apparatus

During Autoshaping, the chamber and the infrared photobeams must be in the autoshaping configuration: the reward unit is fixed in the center on the same side as the touchscreen inside the chamber and the front beam is divided into two independent beams allowing for measurement of approaches to each side of the touchscreen separately. The autoshaping mask (24.3 × 28 cm) is placed in front of the screen, providing two response windows (two white vertical rectangles of 6.5 × 14 cm) displayed on the left and right of the reward tray. The protocol used was adapted from the training schedule described in^20^.

#### Protocol

Training: After Hab 1 (see above), mice were trained to operate the touch screen. Mice were initially given one 30 min session per day for two consecutive days (Hab 2) in which they were allowed to habituate to the testing chamber and collect the reward (strawberry milkshake) from the central food magazine. The house light was illuminated, and reward was delivered into the central magazine on a variable intertrial interval (ITI) 10–40 sec schedule. Notice, before the beginning of each session, a bolus of milkshake was delivered to familiarize mice with the reward.

*Test*: One day after Hab 2, mice began the autoshaping task where they learn to associate one side of the screen as positive conditioned stimulus (CSp) and the other side as negative conditioned stimulus (CSn). The CSp signals the delivery of 7 µl milkshake immediately after the offset of the stimulus, associated with a tone and the illumination of the tray light. No tone or reward occurs upon CSn display. One trial consists of the presentation of CSp or CSn in a randomized order during 10 seconds. After stimulus presentation and entrance to the magazine for reward collection if CSp displayed, an inter-trial interval (ITI) variable begins (10–40 s), after which the mouse is required to break the infrared beam at the rear of the chamber causing initiation of the next trial, thus reducing chance approaches and ensuring equal stimulus sampling. When a mouse breaks either the left or right infrared beam that runs either side of the central food magazine, it is scored as an approach to that stimulus, and no additional approaches are scored under “lit CS” during that stimulus presentation. If the first approach during a trial is to the unlit screen, it falls under the “total CS” category. All additional approaches during before and after a trial fall under the “all CS” category. Stimulus presentation is performed in pairs, such that within a 40 trial session, there are 20 presentations of each CSp and CSn. CSp and CSn side selection is counterbalanced across subjects.

### 5-Choice Reaction Time Task (5-CSRTt)

#### Apparatus

During 5-CSRT, the chamber and the infrared photobeams are set in the “normal” configuration: the reward dispenser is fixed on the opposite side to the touchscreen. The 5-CSRT mask (24.3 × 28 cm) is placed in front of the screen, providing five response windows (small squares of 3 × 3 cm). Stimulus displays are represented as a white square in dimensions similar to the response windows. The protocol used was adapted from the training schedule described in^20^.

#### Protocol

Training: Given the complexity of the task, a 4-step training is required before the mice can reach testing phase. Criteria are set for each step and must be reached for a mouse to be able to move to the next step. In step I, mice are initially given 2 sessions (30 min), in which they are allowed to habituate to the testing chamber and collect milkshake from the food magazine. Once mice are reliably retrieving and consuming the reward (30 trials in 30 min), they can move to the next phase. In step II called “Initial Touch”, mice are trained to detect a brief visual stimulus presented randomly in 1 of the 5 spatial locations. After a delay, the image is removed and strawberry milkshake is delivered accompanied by illumination of the reward light. Collection of the reward turns off the reward light and the ITI begins (5 s), after which another stimulus is presented. If the mouse touches the stimulus while it is being displayed, the image is removed and three times as much reward is delivered immediately in order to reinforce the mouse’s correct touching behavior. This is repeated until the 30 trials in 60 min criterion is reached. In step III or “Must Touch”, mice have to touch the stimulus in order to obtain reward delivery. The criterion is 30 trials per 60 min. In step IV or “Must Initiate”, mice are expected to initiate the next trial by entering and exiting the reward tray with their heads. The rest of the session was similar to step III. Once the criterion has been reached for two consecutive sessions, the animals are allowed to proceed to baseline 5-CSRT testing.

*Test*: In baseline testing, the first trial is initiated when the mouse collects the reward from the food magazine. After a fixed 5 s intertrial interval (ITI), the stimulus appears in one of the windows for a short period (32 s). Responses in this aperture during illumination and for 5 s afterwards (the limited hold period) are rewarded with the delivery of a single reward dose, and a correct response is recorded. Responses in a non-illuminated hole during the signal period (incorrect response) and failures to respond within the limited hold period (omission) result in a timeout period during which the house light is on for 5 s. Responses in the apertures during the ITI are recorded as premature responses and also result in a timeout period. Additional responses in the apertures during the limited hold period following a correct response are recorded as perseverative responses. A response in the food magazine after the delivery of the reward, or after the timeout period, initiates the next trial. The 5-CSRT session is repeated until animals complete 40 trials/session and achieve >80% accuracy and <20% omissions on two consecutive days. When the criteria are met, the animal is advanced to the next test phase which involves reduced stimulus duration to test for deficits in attentional accuracy.

### Statistical analyses

Independent groups of animals were used for each experimental series (Imaging, self-administration, touchscreen/autoshaping and touchscreen/5-CSRT). Depending on the figure, data were analyzed using appropriate statistical test, including two-tailed unpaired t-test, two-way analysis of variance (ANOVA) or two-way repeated measures ANOVA. Significant main effects and/or interactions of the ANOVAs were further investigated with the Bonferroni’s post hoc test or method of contrast analysis. Data are expressed as mean ± s.e.m. Statistic summary for each figure are shown in Supplemental information.

## Results

### MOR knockout mice show weakened functional connectivity between the prefrontal cortex (PFC) and the nucleus accumbens (NAc)

Our previous Rs-fMRI neuroimaging study revealed predominant FC alterations in reward/aversion networks upon deletion of the MOR gene^6^. Here, we undertook a hypothesis-based approach using the same Rs-fMRI datasets, and focused on the PFC in order to determine whether this brain area shows any FC modification with the rest of the brain. We generated the PFC seed using the 2011 Allen Mouse Brain Connectivity Atlas (Fig. 1A), computed FC of the seed with all the voxels of the brain (voxelwise) for each subject individually, then performed group comparison (MOR knockout versus control) (see methods). Correlation analysis showed significant differences, mainly in frontal areas (Fig. 1B) and, notably, all the significant modifications showed reduced FC (MOR knockout < control), indicating that PFC connectivity overall is weakened in mutant mice.

Among these changes, we identified six anatomical regions where most changes occur, i. e. the piriform area, the substantia innominata, the NAc, the somatosensory cortex, the orbitofrontal cortex and the somatomotor areas. To quantify the reduction of PFC connectivity with these regions, we generated seeds for each target region using the 2011 Allen Mouse Brain Connectivity Atlas (Fig. 1C), as done for the PFC. We counted either the percent of voxels within the target region that show significant FC reduction with PFC (Fig. 1D,E) or the total number of voxels showing significance difference within the target seed (Fig. 1E). The two quantification methods concurred to indicate that the PFC displays highest reduction of correlated activity with the NAc (23% of NAc voxels corresponding to 449 NAc voxels overall).

Together, this analysis demonstrates that PFC connectivity is reduced with several brain regions, and that predominant modifications take place in the NAc, suggesting that the PFC-NAc communication is impaired upon deletion of the MOR gene. To challenge motivated behavior related to PFC-NAc activity, we then used a classical operant self-administration approach, and also designed two behavioral tasks in the TouchScreen.

### MOR knockout mice show decreased motivation for palatable food in a classical operant-self administration procedure

We first tested whether motivation to obtain palatable food could be detected using a classic nose-poke operant paradigm. MOR−/− mice were measured for operant responding to highly palatable chocolate-flavored pellets under a fixed ratio- 1 (FR1) and 3 (FR3) schedules. Our data show significant delayed latencies to reach criterion for both FR1 and FR3 and decreased seeking behavior during an extinction session (Supplementary Result and Supplementary Fig. S2). These initial results demonstrated an altered motivational state in MOR knockout mouse strain. We then undertook touchscreen-based behavioral tasks to study both motivational and cognitive aspects of this phenotype in great details.

### MOR knockout mice show delayed learning in the autoshaping task (training phase)

**Figure 2:**
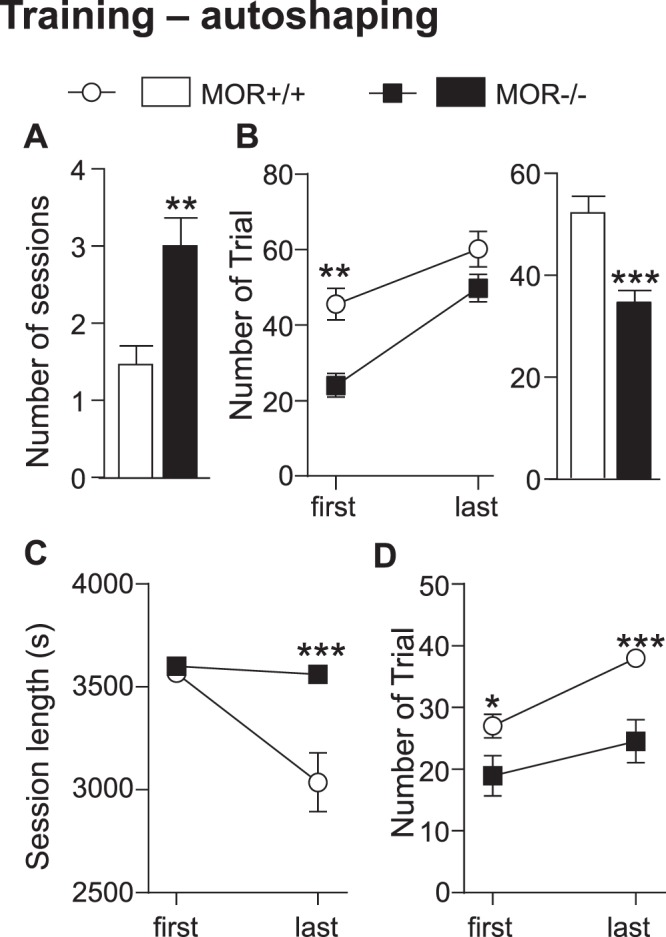
MOR−/− mice show impaired acquisition of stimulus-reward association in the autoshaping task. Mice were tested in the TouchScreen autoshaping paradigm composed of a training and a testing phase. **AB**. *Training phase*. The average number of sessions is higher (**A**), the average number of trials per session for first and last session (B left panel) and the average number of trials per session across all the training sessions (B right panel) are lower in mutant mice**. CD**. *Testing phase*. Session length (**C**) is reduced during the last session of this phase in control animals but not in MOR−/− and number of trials (**D**) is diminished in MOR−/− during the first and last session compared to MOR+/+ mice. Data are expressed as mean ± s.e.m. N = 10–13, *p < 0.05; **p < 0.01; ***p < 0.001. Detailed statistics are shown Supplementary Table S2.

The first step of this task is a training phase that consists in learning the association tray-visual cue-apparition of a reward. As shown in Fig. 2A, MOR−/− mice spent a significantly higher number of sessions to reach criterion in this phase compared to MOR+/+ mice (t_(21)_ = 3.64, p < 0.01). Two-way ANOVA for trials per first vs. last session indicated significant effects of session (Fig. 2B. left, F_(1,21)_ = 1.62, p < 0.001) and genotype (F_(1,21)_ = 20.92, p < 0.001) but no interaction. Subsequent analysis using method of contrast revealed an effect for first (p < 0.01) but not last session, suggesting that MOR−/− mice learn, but slowly compared to their controls. Consequently, the average number of trials over the training phase is significantly reduced in MOR−/− (Fig. 2B. right, t_(21)_ = 4.05, p < 0.001). At the end of the training phase, all animals of both genotypes reached criteria (40 trials per session of 40 min) and were able to move to the testing phase.

During this phase, mice have to learn the stimulus-reward association. As shown in Fig. 2C, two-way ANOVA for session length for first vs. last session revealed significant effects of genotype (F_(1,21)_ = 15.42, p < 0.001), session (F_(1,21)_ = 10.79, p < 0.01) and interaction (F_(1,21)_ = 8.01, p < 0.05). Bonferroni post-hoc analysis showed a significant genotype effect for last (p < 0.001) but not first session. In addition, two-way ANOVA for trials numbers during first vs. last session revealed genotype (Fig. 2D, F_(1,21)_ = 15.42, p < 0.001) and session (F_(1,21)_ = 15.6, p < 0.001) effect but no interaction. Subsequent analysis using method of contrast showed a significant decrease in number of trials for MOR−/− during the first (p < 0.05) and last session (p < 0.001) compared to MOR+/+ mice. Altogether, all animals reached the test phase of the autoshaping task but a lower number of MOR−/− than MOR+/+ mice reached criterion during test phase (40 trials per session of one hour), indicating altogether that the learning performance is lower in mutant mice. Subsequent analysis only includes mice that have reached criterion during the autoshaping test phase.

### MOR knockout mice show normal discriminatory behavior in the autoshaping task (test phase)

**Figure 3:**
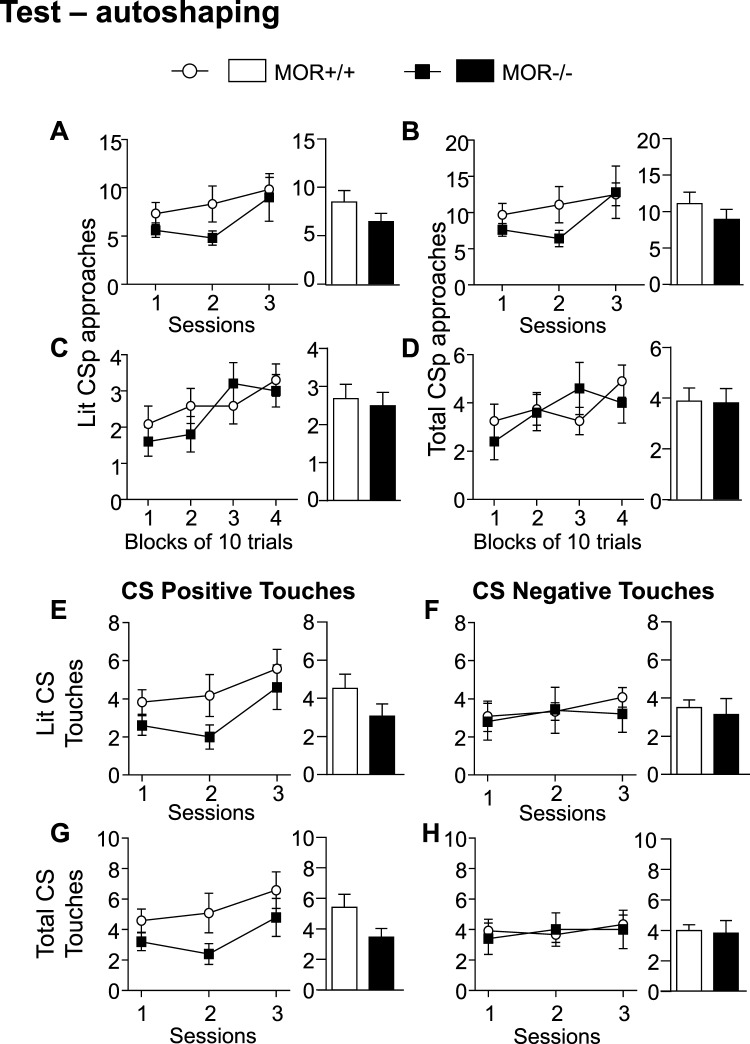
MOR−/− mice show no alteration of discriminatory behavior in the autoshaping task. MOR−/− and their corresponding control animals that reached criterion (40 trials per session) during the autoshaping test phase exhibited comparable discriminatory behavior i.e. increased behavior towards CSp while CSn touches and approaches were stabilized. **I.AB**. Evolution (A and B left panels) and average (A and B right panels) of lit CSp (**A**) and total CSp (**B**) approaches across sessions were similar for both genotypes. Evolution and average of all CSp approaches were also similar across sessions (see Supplementary Fig. S3A). **I.CD**. Evolution (C and D left panels) and average (C and D right panels) of lit CSp (**C**) and total CSp (**D**) approaches across 10-trial blocks during the first session were similar for both genotypes. Evolution and average of all CSp approaches were also similar across 10-trial blocks (see Supplementary Fig. S3C). **II.AB**. Evolution (A and B left panels) and average (A and B right panels) of lit CSp (**A**) and CSn (**B**) touches across sessions were similar for both genotypes. Evolution and average of all CSp and all CSn touches were also similar across sessions (see Supplementary Fig. S3B and D). **II.CD**. Evolution (C and D left panels) and average (C and D Right panels) of lit CSp (**C**) and CSn (**D**) approaches across 10-trial blocks during the first session were similar for both genotypes. Data are expressed as mean ± s.e.m. N = 5–12. Detailed statistics are shown in Supplementary Table S3.

During the test phase of an autoshaping task, mice usually escalate the number of approaches or touches in response to CSp while the number of approaches and touches associated with the CSn stabilizes. In order to evaluate the effect of the MOR gene knockout on discriminated behavior, we analyzed the number of approaches and touches for either CSp or CSn in mice that had reached the test criteria (40 trials per session of one hour during testing phase). Here, we show the results for all three sessions as well as the average per block of ten trials during the first session. Both MOR+/+ and MOR−/− mice increased their CSp approaches across the first three sessions (Fig. 3A,B left) as well as across the blocks of the first session (Fig. 3C,D left). Two-way ANOVA with repeated measures (RM) for approaches per session revealed a significant session effect for total CSp approaches (defined as the total number of approaches towards the CSp when either the CSp or the CSn is lit, Fig. 3B left, F_(2,30)_ = 3.24, p = 0.05) suggesting that mice from both groups reaching criterion during training phase learn to associate the CSp to the reward. No significant session effect on number of lit (defined as the number of approaches specifically towards the CSp when the CSp is lit Fig. 3A. left) and all CSp approaches were detected. Also, no genotype or interaction effects were found significant for any tested parameters. Two-way ANOVA with RM performed on number of approaches per block of 10 trails during the first session revealed a significant block effect for lit CSp (Fig. 3C left, F_(3,45)_ = 3.54, p < 0.05) strengthening the idea that, when reached criterion, MOR+/+ and MOR−/− mice learned the stimulus-reward association similarly. No significant session effect on number of total CSp (Fig. 3D left) and all CSp (Supplementary Fig. S3A,C) approaches were detected. Also, no genotype or interaction effects were found significant for any tested parameters and the average of lit and total CSp across sessions and blocks were similar in both genotypes (Fig. 3A–D right).

Equally, MOR+/+ and MOR−/− mice showed a tendency to increase touches of the CSp across sessions while stabilizing touches of the CSn. The average of touches for Lit CSp, total CSp and all CSp were similar in both genotypes across the sessions. Two-way ANOVA with RM performed on number of touches over sessions showed a significant session effect for Lit CSp touches (Fig. 3.E left, F_(2,30)_ = 3.58, p < 0.05). No significant session effect on number of total (Fig. 3G left) and all CSp touches (Supplementary Fig. S3B) were detected. Also, no significant effects for genotype or interaction for any of the three parameters were detected. MOR+/+ and MOR−/− also showed similar numbers of lit, total and all CSn touches (Fig. 3F,H right as well as Supplementary Fig. S3D). Two-way ANOVA performed on touches per session showed no effect of time, genotype or interaction (Fig. 3F,H). Our analysis therefore suggests that MOR+/+ and MOR−/− mice do not differ during the test phase.

In sum, data from the autoshaping task indicate that MOR−/− animals show a lower performance in the acquisition of stimulus-reward association compared to controls during the training phase. Mutant animals that reached criterion further escalated their behavior towards CSp while stabilizing behavior in response to CSn similarly to controls, indicating that learning is delayed but CS discrimination is preserved in MOR−/− mice.

### MOR knockout mice show delayed learning in the 5-CSRT task (training phase)

The 5-CSRT, an established methodology that mainly explores attention, inhibitory control, and perseveration, can also provide measures of learning, as the cognitive complexity of the test requires a long training period before mice can undergo the test^21^. This training is divided into four main steps and can last as many sessions with: as it takes (with one session per day) for the animal to reach the criteria (40 trials on two out of three consecutive days) and move to the next step.

**Figure 4:**
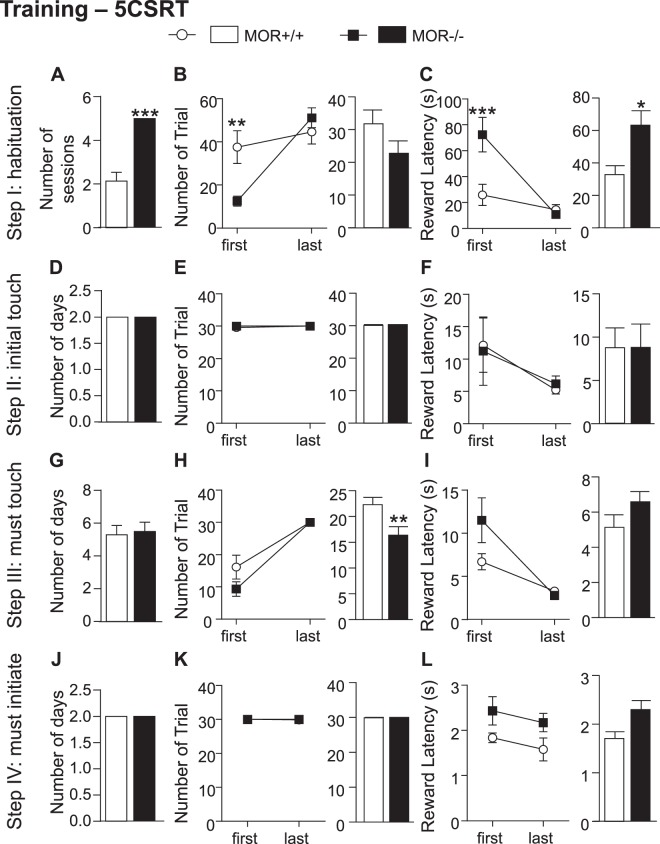
Learning is delayed and reward value is decreased for MOR−/− mice in the 5-Choice Serial Reaction Time task (5-CSRT). MOR−/− showed impaired acquisition of stimulus-reward association during the habituation part of the training phase. *Step I*. (**A**) Average of number of sessions to reach criterion is higher in mutant mice. (**B**) Number of trials per session is reduced in MOR−/− during the first session (left panel). No difference observed between both genotypes on number of trials during last session and on the average of trials (right panel) per session across all sessions. (**C**) Reward latency for first session (left panel) is reduced in MOR−/− mice leading to a decrease in the average of reward latency per session across all step I sessions (right panel). *Step II-IV*. **DGJ**. Average of number of sessions is similar for MOR−/− and control animals for Initial touch (**D**), Must touch (**G**) and Must initiate (**J**). **EHK**. Number of trials per session for first and last session (left panels) as well as average of trials per session across all sessions (right panels) of Initial touch (**E**), Must touch (**H**) and Must initiate (**K**) phases are not altered in mutant mice. Evolution of number of trials per session for each mouse during the Must Touch phase is shown in (**B**) **FIL**. Reward latency for the first and last session (left panels) in addition to average of reward latency per session across (right panels) of Initial touch (**F**), Must touch (**I**) and Must initiate (**L**) phases are not altered in mutant mice. Overall the total number of sessions to reach criterion during training phases and move to test is significantly higher in MOR−/− (See Supplementary Fig. S4A,B). Data are expressed as mean ± s.e.m. N = 7–8, *p < 0.05; **p < 0.01; ***p < 0.001. Detailed statistics are shown Supplementary Table S4.

In the habituation phase (step I), a first observation was that the total number of sessions required for training was significantly increased in MOR−/− compared to MOR+/+ mice (Supplementary Fig. S4A, t_(11)_ = 2.85, p < 0.05), indicating that overall knockout animals take longer to reach criteria and move on to the test. The analysis of each training step, further indicates that the difference of total number of sessions is due to a significant genotype effect in the first step of the training (Fig. 4A, t_(11)_ = 6.5, p < 0.0001). In this habituation phase, similar to the autoshaping training phase, mice learn to associate the tray to the apparition of a reward. A two-way ANOVA for number of trials during the first vs. last session revealed a significant main effect of sessions (Fig. 4B left, F_(1,22)_ = 7.82, p < 0.001), no effect of genotype and a significant interaction (F_(1,22)_ = 2.66, p < 0.05). Bonferroni Post hoc analysis showed significant decrease of trial counters at first (p < 0.01) but not last session. However, there was no significant effect on the total number of trial counter over all sessions (Fig. 4B right), suggesting that MOR−/− mice learn, though at a slower rate.

Next, we tested whether the difference of trial counters impacts latency to collect reward. As shown in Fig. 4C left, two-way ANOVA for reward latency during the first vs. last session of the training phase revealed significant effects of session (F_(1,11)_ = 15.78, p < 0.01), genotype (F_(1,11)_ = 10.84, p < 0.01) and interaction (F_(1,11)_ = 7.44, p < 0.05). Similar to trial counter, Bonferroni post hoc analysis showed significant decrease of trial counters at first (p < 0.001) but not last session. Interestingly in this case, the average total latency to collect reward was also significantly increased in MOR−/− mice (Fig. 4C right, t_(37)_ = 2.52, p < 0.05) compared to MOR+/+ mice. As for the autoshaping task, therefore, MOR−/− mice show delayed learning during the habituation phase, and the higher latency to collect reward further suggests decreased reward value in these animals.

In the three other training steps, we observed no genotype difference, except for the number of trial counters in step III (Fig. 4H right, t_(70)_ = 2.76, p < 0.01), the “Must Touch” phase in which mice have to touch the stimulus in order to obtain a reward. This could be due once again to a delay to reach criterion in a step that is complex and the acquisition of which takes up to 8 days (see Supplementary Fig. S4B). No significant genotype effect was observed for step II (Fig. 4D–F) or step IV (Fig. 4J–L).

In fine therefore, all the mice learned the task and reached criteria to move to test phase. Similarly to autoshaping training, however, MOR−/− mice showed delay in learning during the habituation step, and this delay was associated with a reduction in reward value.

### MOR knockout mice show intact attention in the 5-CSRT task (test phase)

The 5-CSRT test delivers information on accuracy, a measure of attention. Accuracy of performance was measured as the proportion of correct responses (correct responses/total responses) expressed as a percentage, without including errors of omission. Because each mouse spends a specific number of sessions per interval, the results are presented per interval rather than per session, an interval being defined by the time of appearance of the stimulus (32, 16, 8, 4 or 2 seconds) during a given number of intervals until criteria (>80% accuracy, <20% omission) are reached.

**Figure 5:**
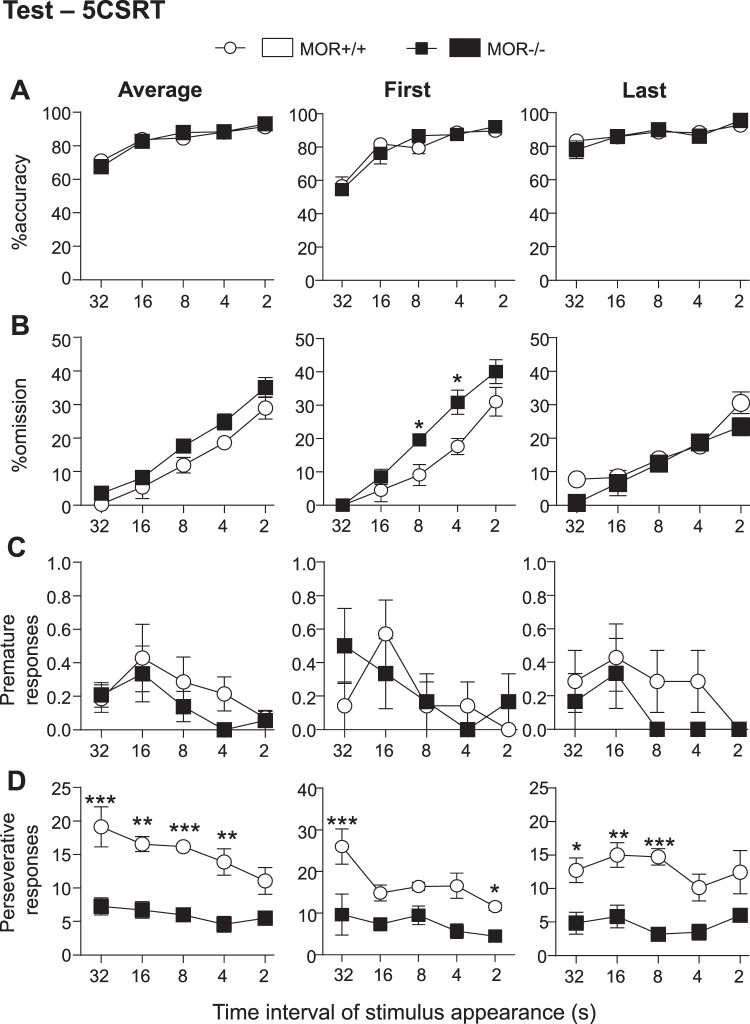
MOR−/− mice show intact attention but lower motivation and perseveration in a 5-CSRT test. MOR−/− mice showed a preserved attention processes and significantly reduced compulsive-like behavior in the 5-CSRT task. (**A**) % of accuracy across different intervals of stimulus is similar for both genotypes. Data represent average of % of accuracy for all, first and last sessions for each interval. (**B**) MOR−/− mice show an increase of % omission in the first session of each interval compared to control mice and this effect disappear during the last session of every phase. Data represent average of % omission for all, first and last sessions for each interval. (**C**) No difference between MOR−/− and their control mice on the number of premature responses. Data represent the average of the number of premature response during all, first and last sessions for each interval. (**D**) MOR−/− mice showed a drastic decrease of perseverative responses in comparison to MOR+/+ animals. Data represent the average of the number of perseverative response during all, first and last sessions for each interval. In addition, the evolution of both front and back beam breaks across different intervals of stimulus appearance was similar in both genotypes (see Supplementary Fig. S5A,B). Overall, number of sessions per interval to reach criteria as well as average number of sessions to finish the 5-CSRT test was similar across genotypes (see Supplementary Fig. S5C,D). Data are expressed as mean ± s.e.m. N = 7–8, *p < 0.05; **p < 0.01; ***p < 0.001. Detailed statistics are shown in Supplementary Table S5.

Two-way ANOVA with RM for the average of accuracy percentage per interval revealed a main effect of interval (Fig. 5A, F_(4,55)_ = 40.42, p < 0.0001) but no effect of genotype and no interaction, meaning that MOR+/+ and MOR−/− mice display similar percentage of accuracy. Performing a two-way ANOVA with RM on first (Fig. 5A, F_(4,55)_ = 33.66, p < 0.0001) or last (Fig. 5A, F_(4,55)_ = 8.66, p < 0.0001) session of each interval also showed a main effect of interval but no effect of genotype or interaction. Altogether therefore attention processes seem intact in MOR−/− mice, at least as measured by the 5-CSRT task.

### MOR knockout mice show decreased motivation during the 5-CSRT task (test phase)

Errors of omission were defined as failures to make a response during the 5 s limited hold period, expressed as a percentage of the total number of trials. This measure reflects possible failures of detection as well as motivational/motor deficits, depending on the overall pattern of effects^22^.

Two-way ANOVA with RM for the average of omission percentage per interval revealed a significant effect of interval (Fig. 5B, F_(4,55)_ = 54.04, p < 0.0001) and genotype (F_(4.55)_ = 10.88, p < 0.01) but no interaction. Performing a two-way ANOVA with RM on first (Fig. 5B, F_(4,55)_ = 47.38, p < 0.0001) or last (Fig. 5B, F_(4,55)_ = 33.43, p < 0.0001) session of each interval confirmed the effect of interval. However, the genotype effect was only present in the first session of each interval (F_(4,55)_ = 16.03, p < 0.001), not the last. There was no interaction in either the first or the last sessions. MOR−/− mice thus show an increase of % omission in the first session of each interval compared to control mice and this is compensated at the last session of every phase.

An increase of response omissions in the absence of accuracy change is regarded as an indicator for decreased motivation and/or motor deficit i.e. a goal that stimulates an action^22^. As an indicator of both motor activity and goal-directed behavior, we measured the front and back beam breaks, the front being the screen beam (sign) and the back being the reward tray beam (goal). Two-way ANOVA showed a significant decrease of back beam breaks in MOR−/− compared to MOR+/+ mice with a main effect of genotype (Supplementary Fig. S5B). F_(1,55)_ = 11.3, p < 0.01) but no effect of interval and no interaction. There was no genotype difference for the front beam breaks (Supplementary Fig. S5A). Together therefore, data showing increased omission with intact accuracy in the first session strongly suggest decreased motivation for the reward in MOR−/− mice, and is in line with decreased reward value detected in the autoshaping task.

### MOR knockout mice show decreased perseverative responses in the 5-CSRT task (test phase)

As previously reported, MORs normally facilitate motor impulsivity, based on the observation that MOR−/− mice perform remarkably well in a signaled nose poke task^15^. 5-CSRTt also provides a measure of motor impulsivity (premature responses). This task additionally provides a measure of compulsivity (perseverative responses) that is relatively outcome-insensitive, as opposed to the habitual nature of relatively incentively motivated impulsive action^23^. Perseverative responses is defined as continued screen touches after a correct response has been performed.

Given the very low number of premature responses recorded for our control mice, we did not observe significant reduction of motor impulsivity in MOR−/− mice as previously reported^15^. However, there was a trend despite the limiting factor (see Fig. 5C). Interestingly, we observed a drastic decrease of perseverative responses in MOR−/− mice compared to MOR+/+ animals (Fig. 5D). Two-way ANOVA performed on genotype per interval revealed a main genotype effect in the average of sessions per interval (Fig. 5D, F_(1,55)_ = 79.67, p < 0.0001). This result was similarly strong for the first (Fig. 5D, F_(1,55)_ = 39.59, p < 0.0001) and last (Fig. 5D, F_(1,55)_ = 50.76, p < 0.0001) session of each interval. Two-way ANOVA also revealed an interval effect for the average of sessions per interval (Fig. 5D, F_(4,55)_ = 2.78, p < 0.05) but not for the first or last sessions of each interval. There was no genotype effect per interval interaction.

Altogether therefore, MOR−/− mice showed significantly reduced perseverative behavior in the 5-CSRT task, a novel finding that can be interpreted as lower compulsive-like behavior.

## Discussion

Here we combined PFC seed-based FC analysis by MRI, operant self-administration procedure and Touchscreen-based autoshaping/5-CSRT tasks to investigate whether MORs regulate reward-driven motivated behavior, and related cognitive controls. Our results reveal that constitutive deletion of the MOR gene reduces PFC-NAc connectivity, an alteration concordant with the low motivation to gain a reward observed in MOR knockout mice.

The present TouchScreen-based behavioral study adds support to converging literature indicating that MOR activity facilitates the stimulus/reward association. We found that MOR−/− mice exhibit longer latencies to get the reward within sessions, as well as delayed learning along the training sessions for both autoshaping and 5-CSRT tasks. During these phases, the number of sessions required to reach criterion was higher and trial counts per session were reduced in MOR−/− mice, while session lengths were increased. All these measures of poor learning performance converge to suggest that MOR normally facilitates the stimulus-reward learning rate. Interestingly, when reaching criterion (autoshaping) or over sessions of training (5-CSRT), MOR −/− mice performed the task similarly to control animals, indicating that MOR specifically contributes to the acquisition phase of the stimulus/reward association.

Deficient stimulus/reward association in MOR−/− mice could be due lower hedonic value of the reward (strawberry milkshake), at least in part. It is well established that MOR plays a key role in mediating both drug and natural rewards (see introduction). Relevant to naturel reward and this study, an earlier report showed decreased consumption of sweet fluids in MOR−/− mice, suggesting altered hedonic value of the natural reward in these animals^24^. Also, licks for sucrose and sucralose, a calorie-free substance to study licking behavior independently from homeostatic variables, were decreased in MOR−/− mice, providing direct evidence for MOR contribution to hedonic palatability^24^. Finally, MOR blockade directly in the NAc decreased licking and consumption of palatable solutions, whereas MOR agonists increased these behaviors^25–27^. Lower hedonic value of the reward therefore certainly explains part of the observed phenotypes in our study, notably slow learning of the tasks.

In addition to lower hedonic value of the reward, a lower motivation to get the reward may also account for the deficient stimulus/reward association observed in this study. In support of this other mechanism, we observed reduced number of completed trials during the autoshaping task and first step of 5-CSRT training, as well as increased number of omission during the 5-CSRT, and this behavioral pattern typically reflects reduced motivation for the food reward. Note that this particular phenotype observed in the touchscreen testing system was also detected using classical operant self-administration, where mutant mice showed delayed latency to reach criterion and decreased seeking behavior for palatable food. This proposed role of MOR in motivation is consistent with findings of reduced responding in operant nose poke schedules (FR3 and progressive ratio) for both regular chow and palatable food in MOR mutant mice^8^. Also concordant, mice infused with MOR agonists in the NAc^28^ or the PFC^29^ showed enhanced palatable food-self-administration in a progressive ratio schedule, reflecting enhanced motivation for the reward. In addition, it has been shown that MOR agonists in the striatum enhanced cue-triggered ‘wanting’ for reward assessed by pavlovian instrumental transfer procedure^30^. Moreover, pain-induced loss of MOR function in the mesolimbic pathway has been associated with increased opioid intake and associated motivated behavior leading to opioid addiction phenotypes. Altogether, these findings and others^31,32^ suggest that MOR signaling is also critical in motivated behaviors, a role which also contributes to the altered reward/stimulus association phenotype of MOR mutants.

A second main observation from our behavioral study is the drastic reduction of perseverative responses in the 5-CSRT test phase, which we observed in MOR−/− mice. This result aligns with our previous study showing decreased motor impulsivity in MOR−/− mice, as measured by premature responses in a signaled nose poke task^15^. Although we were not able to detect a similar reduction of premature responses in the present study, as the baseline was too low, the two studies together concur to suggest that MOR activity normally favors both impulsivity^15^ and compulsive-like behavior (this study), a notion that is particularly critical for psychiatric disorders including substance use disorders^33–36^, behavioral addictions^37,38^, eating disorders with binging features^39,40^ or ADHD^41,42^. Our results are in accordance with a previous study conducted in humans, which associates high MOR density and greater stress-induced endogenous opioid system activation in brain regions involved in motivated behaviors (e.g. prefrontal and orbitofrontal cortices) with increased impulsiveness and low deliberation scores^43^.

Previous data from our group showed modifications of whole-brain FC in MOR−/− mice, with predominant reshaping of reward/aversion networks^6^. Here, we further showed that PFC connectivity is reduced with several forebrain structures, with prominent reduction of PFC-NAc connectivity. This observation suggests that MOR activity contribute to the regulation of networks essential for motivational processes and decision-making. As in our initial fMRI analysis^6^, this finding reveals an interesting parallel between MOR knockout mice phenotypes at the level of whole-brain networks (weakened PFC-NAc connectivity) and behavior (dysregulation of stimulus/natural reward association, reduced reward motivation and increased perseveration). Interestingly in humans, lower inhibitory control is generally associated with weaker fronto-striatal functional connectivity^44^, and G-allele carriers for the MOR gene showed stronger negative fronto-striatal functional connectivity during a single alcohol taste-cue trial, compared to the more frequent A-allele carriers^45^.

Our combined MRI and behavior datasets, together, could raise the possibility that MOR in the PFC help setting the motivational drive to obtain a reward. In support of this hypothesis, previous literature showed that in human ventromedial prefrontal cortex (vmPFC) play a major role in reward evaluation and specifically in decision-making with known outcome probabilities^46^ and in mice PFC-NAc neurons orchestrate reward-learning by encoding reward-predictive stimuli^47^. This reward representation in turn facilitates decision making and depends on the motivational state of the subject^48^. MORs may be players in this complex process to enhance reward learning and seeking, and further targeted MOR deletion or rescue within PFC neurons and circuits will establish whether this hypothesis is valid.

In conclusion, the broad link between reward/motivation and cognitive functions is complex^49,50^ and our study definitely positions MORs at the center of these processes. Beyond the traditional view of a main role for MORs in mediating the hedonic value of drug and natural rewards, our study reveals a role for the receptor in more integrated facets of behavior, including the facilitation of reward-based motivation and learning, as well as the promotion of compulsive behavior. Regarding the later, the notion that blocking MOR activity may not only to limit drug reward but also reduce maladaptive habit forming is highly novel in the area of SUDs.

## Supplementary information

Supplementary Information
