# Supplementary material for: TouchScreen-based phenotyping: altered stimulus/reward association and lower perseveration to gain a reward in mu opioid receptor knockout mice
Source: Sci Rep. 2019 Mar 11;9:4044. doi: 10.1038/s41598-019-40622-6 (PMC6411729; doi:10.1038/s41598-019-40622-6)
Supplement: Supplementary file 1 — Supplementary Information [file 41598_2019_40622_MOESM1_ESM.docx]

**Supplementary Information**

**TouchScreen-based phenotyping: altered stimulus/reward association and lower perseveration to gain a reward in mu opioid receptor knockout mice**

Laura-Joy Boulos^1^, Md. Taufiq Nasseef^1^, Michael McNicholas^1^, Anna Mechling^2^, Laura Adela Harsan^2, 3, 4^, Emmanuel Darcq^1,^, Sami Ben Hamida^1,5^ and Brigitte Lina Kieffer^1, 5*^

^1^Douglas Mental Health Institute, Department of Psychiatry, McGill University, Montreal, Quebec, Canada.

^2^Department of Radiology, Medical Physics, Medical Center University of Freiburg,

Faculty of Medicine, University of Freiburg, Freiburg, Germany

^3^Engineering science, computer science and imaging laboratory (ICube), Integrative

Multimodal Imaging in Healthcare, University of Strasbourg – CNRS, Strasbourg,

France

^4^Department of Biophysics and Nuclear Medicine, Faculty of Medicine, University

Hospital Strasbourg, Strasbourg, France

^5^These authors jointly directed the work

**Supplementary Result**

**MOR knockout mice show decreased motivation for palatable food in a classical operant-self administration procedure.**

We tested whether motivation to obtain palatable food could be detected using a classic nose-poke operant paradigm that measures motivational processes. Specifically, we assessed the role for the MOR in the motivational properties of food intake nose-poke operant paradigm. MOR^-/-^ mice were measured for operant responding for highly palatable chocolate-flavored pellets under a fixed ratio- 1 (FR1) and 3 (FR3) schedules (See Supplementary Methods below). We measured the latency to reach 10 rewards during the last day under FR1 and 10 or 25 rewards during the last day of FR3. We observed significant delays in reaching criterion in MOR^-/-^ mice compared to control group (10 rewards FR1; Fig. S2A: t_(26)_ = 2.97, p < 0.01; 10 rewards FR3; Fig. S2B: t_(26)_ = 3.7, p < 0.001; 25 rewards FR3; Fig. S2C: t_(26)_ = 2.1, p < 0.05 ; only mice that reached criterion were included in the analysis). This data suggest a decreased motivation to seek highly palatable reward in MOR mutant mice. To confirm this possibility, instrumental performance was analyzed during an extinction session in which reward was not delivered upon nosepokes. As shown in Fig. S2D, decreased reward-seeking as reflected by a reduction in the number of nosepokes during an extinction session was detected (t_(28)_ = 2.54, p = 0.01) suggesting an altered motivational state of mutant animals. Together these findings confirm the behavioral phenotype observed in the touchscreen tasks and indicate that the MOR pathway mediates the motivational properties of highly palatable food intake.

**Supplementary Figures**

**A**

**Hab 1**

handling, weighing,

food deprivation

**Autoshaping**

**test**

**Hab 2**

Acclimatise to the apparatus and become familiar with the reward

Hab 1

Mice must reach 80-85% of free feeding body weight before Hab2

Hab 2

Must have 40 trials presented in 30 minutes for 2 days before moving onto the test phase

Test

Must respond correctly to 40 trials in 60 minutes

**Training Step I**

**Step III**

must touch

**Step IV**

initiate touch

**Step II**

Initial touch

**5-CSRT**

**test**

Step II

Step III

Correct response to >30 trials in 60 minutes

Test

Step IV

>30 trials in 60 minutes presented

Initiate >30 trials in 60 minutes

Advancing to a shorter stimulus length requires Accuracy >80% and Omission <20% for two consecutive days.

**B**

Premature nosepoke

ITI Interval

Start Delay

End Delay

Collect reward

Enter/exit food tray

Stimulus presentation

Omission

Reward

TOUCH

TOUCH

No

T.O

Yes

Incorrect

Correct

ITI Interval

Collect reward

Trial Initiated

Reward

CSp Display

CSn Display

Stimulus removed

Stimulus presentation

**Supplementary Fig. S1- Touchscreen protocols-** All procedures are accomplished in standard mouse Bussey-Saksida Touchscreen system. **A. Autoshaping protocol.** The autoshaping protocol consists of a training and a testing phase (top panel). *Training phase*- The training phase is composed of a pre-habituation phase “Hab 1” i.e. handling and food deprivation (5 days) and a habituation phase named “Hab 2” i.e. learning to associate the food tray to an apparition of the stimulus. Once mice reach criterion, they move to test. *Testing phase*- The testing phase is detailed in the bottom panel, with the flowchart overview of associative learning. Trial is initiated when the mouse inserts its head in the food tray. A stimulus is presented on one of the two sides of the screen. One side (CSp) is always followed by the apparition of a reward in the tray whereas the other is not followed by a reward (CSn). If CSp is displayed and the mouse collects its reward, an inter-trial interval (ITI) starts, at the end of which another trial starts. This loops back until mice reach criterion. **B. 5-Choice Serial Reaction Time task (5-CSRTt) protocol.** The 5-CSRTt protocol consists of a training and a testing phase. *Training phase*- The training phase is composed of 4 steps. Step I named “habituation” is similar to autoshaping training (Hab 1 + Hab 2). Step II named “Initial Touch”, Step III named “Must Touch” and finally in Step IV name “Must Initiate”. *Testing phase*- Once mice reach criterion, they move to the 5-CSRTt test. During test phase, mice have to initiate trial and touch the correct screen in order to obtain a reward. After collecting the reward, animals initiate the next trial then wait during an ITI interval then loop back. However, premature, incorrect and omission responses are punished by a time out (5-second time out during which house light is on and nothing can happen).

0

100

200

300

400

500

Latency to 10 Reward (FR1)

Latency to 10 Reward (FR3)

0

500

1000

1500

Latency to 25 Reward (FR3)

******

*******

*****

0

100

200

300

400

500

**A**

**B**

**C**

0

20

40

60

Number of Nosepokes

(extinction session)

**D**

*****

MOR+/+

MOR-/-

**Supplementary Fig. S2. *MOR* knockout mice show decreased seeking behavior.** MOR-/- mice and their controls were tested for operant self-administration of chocolate flavor pellets does not differ between. Animals were trained under a FR1 and FR3 schedules of reinforcement in 20 minute daily sessionsReduced latency to reach 10 (**A**) rewards under FR1 or to reach 10 (**B**) and 25 (**C**) rewards under FR3 were also observed. **A-C** only animals that reached the criterions were included in the analysis. **D**. Number of presses on the alcohol lever during a 5-min extinction session (reward not available). Data are mean ± SEM. *p < 0.05; **p < 0.01; ***p < 0.001 compared with control group. **A-B** N = 13-15; **C** N =15-12; **D** N = 14-16 for each group.

100

150

All CSp approaches

1

2

3

0

100

200

300

400

1

2

3

4

0

50

Blocks of 10 trials

1

2

3

0

200

400

600

800

1000

1

2

3

0

500

1000

1500

Sessions

All CSp Touches

Sessions

Sessions

All CSn Touches

**C**

**A**

**D**

**B**

**Supplementary Fig. S3- MOR knockout does not impair discriminated behavior in an autoshaping test.** All mice that reached criterion (40 trials per session) during the autoshaping test phase exhibited discriminated behavior i.e. increased behavior towards CSp while CSn touches and approaches were stabilized. **A. left-** Evolution of all CSp approaches across sessions. **A. right-** Average of all CSp approaches across sessions. **B. left-** Evolution of all CSp touches across sessions. **B. right-** Average of all CSp touches across sessions. **C. left-** Evolution of all CSp approaches across 10-trial blocks during the first session. **C. right-** Average of all CSp approaches across 10-trial blocks during the first session. **D. left-** Evolution of all CSn touches across 10-trial blocks during the first session. **D. right-** Average of all CSn touches across 10-trial blocks during the first session. Data are expressed as mean±s.e.m. N=5-12.

1

2

3

4

5

6

7

8

0

10

20

30

40

MOR+/+

MOR-/-

**Number of trials**

**Sessions**

**Training**

0

5

10

15

20

*

**Number of sessions**

**A**

**B**

**Supplementary Fig. S4. MOR knockout impairs acquisition in a 5-Choice Serial Reaction Time task (5-CSRTt) training.** Mice were tested in the TouchScreen 5-CSRT paradigm composed of a training and a testing phase. MOR-/- show impaired acquisition of stimulus-reward association mainly during the habituation part of training. *Training phase*. A- Total number of sessions to reach criterion during training phases and move to test. B- *Step III.* Evolution of number of trials per session for each mouse during the Must Touch phase. N=7-8, * p<0.05.

0

2

4

6

0

5

10

15

Number of sessions

32

16

8

4

2

32

16

8

4

2

0

100

200

300

400

500

Front beam breaks

32

16

8

4

2

100

200

300

400

Back beam breaks

test

**C**

**A**

**D**

**B**

Number of sessions

**Supplementary Fig. S5. MOR knockout decreases motivation and impulsivity/compulsivity but preserves attention in a 5-CSRTt test.** MOR-/- mice showed preserved attention but decreased response rate and impulsivity compared to MOR+/+ mice. **A-** Evolution of front beam breaks across different intervals of stimulus appearance (32 seconds, 16 seconds, 8 seconds, 4 seconds and 2 seconds). **B-** Evolution of back beam breaks across different intervals. **C-** Number of sessions per interval to reach criteria. **D-** Average number of sessions to finish the 5-CSRT test. Data are expressed as mean±s.e.m. N=7-8,

**Table S1. Table showing statistical analysis for all panels of Fig. S2.**

| **Figure** | **Statistical test** | **t or F** | **DFn** | **df or Dfd** | **p** |
| --- | --- | --- | --- | --- | --- |
| Fig. S2A | t-test | 3.64 |  | 21 | 0.0015 |
| Fig. S2B | t-test | 4.05 |  | 21 | 0.0006 |
| Fig. S2C | t-test | 3.64 |  | 21 | 0.0015 |
| Fig. S2D | t-test | 4.05 |  | 21 | 0.0006 |

**Table S2. Table showing statistical analysis for all panels of Fig. 2.**

| **Figure** | **Statistical test** | **t or F** | **DFn** | **df or Dfd** | **p** |
| --- | --- | --- | --- | --- | --- |
| Fig. 2A | t-test | 3.64 |  | 21 | 0.0015 |
| Fig. 2B.right | t-test | 4.05 |  | 21 | 0.0006 |
| Fig. 2B.left | 2way ANOVA Interaction | 1.62 | 1 | 21 | 0.22 |
|  | 2way ANOVA Session | 20.92 | 1 | 21 | 0.0002 |
|  | 2way ANOVA Genotype | 16.29 | 1 | 21 | 0.0006 |
| Fig. 2C | 2way ANOVA Interaction | 1.65 | 1 | 21 | 0.21 |
|  | 2way ANOVA Session | 15.6 | 1 | 21 | 0.0007 |
|  | 2way ANOVA Genotype | 15.42 | 1 | 21 | 0.0008 |
| Fig. 2D | 2way ANOVA Interaction | 8.01 | 1 | 21 | 0.01 |
|  | 2way ANOVA Session | 10.79 | 1 | 21 | 0.0035 |
|  | 2way ANOVA Genotype | 11.24 | 1 | 21 | 0.003 |

**Table S3. Table showing statistical analysis for all panels of Fig. 3.**

| **Figure** | **Statistical test** | **t or F** | **DFn** | **df or Dfd** | **p** |
| --- | --- | --- | --- | --- | --- |
| Fig. 3A.right | t-test | 1.06 | 15 |  | 0.3 |
| Fig.3B.right | t-test | 0.81 | 15 |  | 0.42 |
| Fig.3C.right | t-test | 0.29 | 15 |  | 0.77 |
| Fig.3D.right | t-test | 0.09 | 15 |  | 0.93 |
| Fig.3E.right | t-test | 1.18 | 15 | 0.26 |  |
| Fig.3F.right | t-test | 0.45 | 15 | 0.66 |  |
| Fig.3G.right | t-test | 1.4 | 15 | 0.18 |  |
| Fig.3H.right | t-test | 0.22 | 15 | 0.82 |  |
| Fig.3A.left | 2way ANOVA Interaction | 0.5 | 2 | 30 | 0.61 |
|  | 2way ANOVA Session | 2.99 | 2 | 30 | 0.06 |
|  | 2way ANOVA Genotype | 1.12 | 1 | 15 | 0.31 |
| Fig.3B.left | 2way ANOVA Interaction | 0.96 | 2 | 30 | 0.39 |
|  | 2way ANOVA Session | 3.24 | 2 | 30 | 0.05 |
|  | 2way ANOVA Genotype | 0.67 | 1 | 15 | 0.43 |
| Fig.3C.left | 2way ANOVA Interaction | 0.87 | 3 | 45 | 0.4611 |
|  | 2way ANOVA Block | 3.54 | 3 | 45 | 0.02 |
|  | 2way ANOVA Genotype | 0.16 | 1 | 15 | 0.69 |
| Fig.3D.left | 2way ANOVA Interaction | 1.24 | 3 | 45 | 0.31 |
|  | 2way ANOVA Block | 2.07 | 3 | 45 | 0.12 |
|  | 2way ANOVA Genotype | 0.28 | 1 | 15 | 0.87 |
| Fig.3E.left | 2way ANOVA Interaction | 0.28 | 2 | 30 | 0.76 |
|  | 2way ANOVA Session | 3.58 | 2 | 30 | 0.04 |
|  | 2way ANOVA Genotype | 1.4 | 1 | 15 | 0.26 |
| Fig.3F.left | 2way ANOVA Interaction | 0.2 | 2 | 30 | 0.81 |
|  | 2way ANOVA Session | 2.17 | 2 | 30 | 0.13 |
|  | 2way ANOVA Genotype | 1.95 | 1 | 15 | 0.18 |
| Fig.3G.left | 2way ANOVA Interaction | 1.46 | 2 | 30 | 0.25 |
|  | 2way ANOVA Session | 1.98 | 2 | 30 | 0.15 |
|  | 2way ANOVA Genotype | 1.61 | 1 | 15 | 0.22 |
| Fig.3H.left | 2way ANOVA Interaction | 0.17 | 2 | 45 | 0.84 |
|  | 2way ANOVA Session | 0.37 | 2 | 45 | 0.69 |
|  | 2way ANOVA Genotype | 0.3 | 1 | 45 | 0.59 |

**Table S4. Table showing statistical analysis for all panels of Fig. 4.**

| **Figure** | **Statistical test** | **t or F** | **DFn** | **df or Dfd** | **p** |
| --- | --- | --- | --- | --- | --- |
| Fig. 4A | t-test | 6.5 | 11 | < 0.0001 |  |
| Fig. 4D | t-test | NA | NA | NA |  |
| Fig. 4G | t-test | 0.27 | 11 | 0.79 |  |
| Fig. 4J | t-test | NA | NA | NA |  |
| Fig. 4B.right | t-test | 2.1 | 11 | 0.06 |  |
| Fig. 4E.right | t-test | NA | NA | NA |  |
| Fig. 4H.right | t-test | 2.76 | 11 | 0.007 |  |
| Fig. 4K.right | t-test | NA | NA | NA |  |
| Fig. 4C.right | t-test | 2.52 | 11 | 0.016 |  |
| Fig. 4F.right | t-test | 0.0015 | 11 | 0.99 |  |
| Fig. 4I.right | t-test | 1.54 | 11 | 0.13 |  |
| Fig. 4L.right | t-test | 1.49 | 11 | 0.16 |  |
| Fig. 4B.left | 2way ANOVA Interaction | 7.8 | 1 | 22 | 0.01 |
|  | 2way ANOVA Session | 16.25 | 1 | 22 | 0.0006 |
|  | 2way ANOVA Genotype | 2.66 | 1 | 22 | 0.12 |
| Fig. 4E.left | 2way ANOVA Interaction | 0.85 | 1 | 22 | 0.38 |
|  | 2way ANOVA Session | 0.85 | 1 | 22 | 0.38 |
|  | 2way ANOVA Genotype | 0.85 | 1 | 22 | 0.38 |
| Fig. 4H.left | 2way ANOVA Interaction | 2.82 | 1 | 22 | 0.16 |
|  | 2way ANOVA Session | 58.65 | 1 | 22 | < 0.0001 |
|  | 2way ANOVA Genotype | 2.82 | 1 | 22 | 0.16 |
| Fig. 4.Kleft | 2way ANOVA Interaction | 0.85 | 1 | 22 | 0.38 |
|  | 2way ANOVA Session | 0.85 | 1 | 22 | 0.38 |
|  | 2way ANOVA Genotype | 0.85 | 1 | 22 | 0.38 |
| Fig. 4C.left | 2way ANOVA Interaction | 7.44 | 1 | 22 | 0.02 |
|  | 2way ANOVA Session | 15.78 | 1 | 22 | 0.002 |
|  | 2way ANOVA Genotype | 10.84 | 1 | 22 | 0.007 |
| Fig. 4F.left | 2way ANOVA Interaction | 0.06 | 1 | 22 | 0.81 |
|  | 2way ANOVA Session | 2.6 | 1 | 22 | 0.14 |
|  | 2way ANOVA Genotype | 3.072 | 1 | 22 | 0.99 |
| Fig. 4I.left | 2way ANOVA Interaction | 3.13 | 1 | 22 | 0.09 |
|  | 2way ANOVA Session | 16.37 | 1 | 22 | 0.0005 |
|  | 2way ANOVA Genotype | 2.05 | 1 | 22 | 0.17 |
| Fig. 4.Lleft | 2way ANOVA Interaction | 0.0005 | 1 | 22 | 0.99 |
|  | 2way ANOVA Session | 1.3 | 1 | 22 | 0.27 |
|  | 2way ANOVA Genotype | 6.75 | 1 | 22 | 0.16 |

**Table S5. Table showing statistical analysis for all panels of Fig. 5.**

| **Figure** | **Statistical test** | **t or F** | **DFn** | **df or Dfd** | **p** |
| --- | --- | --- | --- | --- | --- |
| Fig. 5A left | 2way ANOVA Interaction | 0.8 | 4 | 55 | 0.53 |
|  | 2way ANOVA Session | 40.42 | 4 | 55 | < 0.0001 |
|  | 2way ANOVA Genotype | 0.02 | 1 | 55 | 0.9 |
| Fig. 5A middle | 2way ANOVA Interaction | 1.06 | 4 | 55 | 0.3 |
|  | 2way ANOVA Session | 33.66 | 4 | 55 | < 0.0001 |
|  | 2way ANOVA Genotype | 0.003 | 1 | 55 | 0.96 |
| Fig. 5A right | 2way ANOVA Interaction | 0.84 | 4 | 55 | 0.5 |
|  | 2way ANOVA Session | 8.66 | 4 | 55 | < 0.0001 |
|  | 2way ANOVA Genotype | 0.19 | 1 | 55 | 0.67 |
| Fig. 5B left | 2way ANOVA Interaction | 0.27 | 4 | 55 | 0.9 |
|  | 2way ANOVA Session | 54.04 | 4 | 55 | < 0.0001 |
|  | 2way ANOVA Genotype | 10.88 | 1 | 55 | 0.0017 |
| Fig. 5B middle | 2way ANOVA Interaction | 1.667 | 4 | 55 | 0.17 |
|  | 2way ANOVA Session | 47.38 | 4 | 55 | < 0.0001 |
|  | 2way ANOVA Genotype | 16.03 | 1 | 55 | 0.0002 |
| Fig. 5B right | 2way ANOVA Interaction | 1.34 | 4 | 55 | 0.27 |
|  | 2way ANOVA Session | 33.43 | 4 | 55 | < 0.0001 |
|  | 2way ANOVA Genotype | 5.4 | 1 | 55 | 0.24 |
| Fig. 5C left | 2way ANOVA Interaction | 0.34 | 4 | 55 | 0.85 |
|  | 2way ANOVA Session | 2.23 | 4 | 55 | 0.08 |
|  | 2way ANOVA Genotype | 1.5 | 1 | 55 | 0.23 |
| Fig. 5C middle | 2way ANOVA Interaction | 1.15 | 4 | 55 | 0.34 |
|  | 2way ANOVA Session | 2.21 | 4 | 55 | 0.08 |
|  | 2way ANOVA Genotype | 0.11 | 1 | 55 | 0.74 |
| Fig. 5C right | 2way ANOVA Interaction | 0.34 | 4 | 55 | 0.85 |
|  | 2way ANOVA Session | 1.7 | 4 | 55 | 0.16 |
|  | 2way ANOVA Genotype | 2.69 | 1 | 55 | 0.11 |
| Fig. 5D left | 2way ANOVA Interaction | 0.98 | 4 | 55 | 0.43 |
|  | 2way ANOVA Session | 2.78 | 4 | 55 | 0.04 |
|  | 2way ANOVA Genotype | 79.67 | 1 | 55 | < 0.0001 |
| Fig. 5D middle | 2way ANOVA Interaction | 1.15 | 4 | 55 | 0.35 |
|  | 2way ANOVA Session | 3.69 | 4 | 55 | 0.11 |
|  | 2way ANOVA Genotype | 39.69 | 1 | 55 | < 0.0001 |
| Fig. 5D right | 2way ANOVA Interaction | 0.65 | 4 | 55 | 0.63 |
|  | 2way ANOVA Session | 0.98 | 4 | 55 | 0.43 |
|  | 2way ANOVA Genotype | 50.76 | 1 | 55 | < 0.0001 |

**Supplementary Material and Methods**

**Animals**

MOR-/- mice lacking mu opioid receptors were produced as previously described^1^ under a mixed background (50% C57B1/6J:50% 129/SvPas) and compared to their wild type littermates (MOR+/+). All mice were males bred at the Author Institutes. Animals were group-housed (3-5 animals per cage) for all the experiments under a 12 h light/dark cycle and received water and food *ad libitum* until one week prior to experiments. During all behavioral experiments, mice underwent a restriction diet, which consisted in providing restricted amounts of standard laboratory chow pellets daily at 6 p.m and maintain all animals at approximately 85% of respective baseline free feeding weight. Drinking water was available ad libitum. All animal procedures were conducted in accordance with the guidelines set forth by the Author Institutes.

**Touchscreen Apparatus**

The test apparatus consists of fiberboard box (25 cm × 25 cm) individually housed within sound-attenuating cabinets and low-level noise fans. Each box comprises a standard operant chamber and a touchscreen (12.1-inch monitor). The ceiling of the chamber is made of clear Plexiglas and the floor of perforated stainless steel with a waste tray situated below. Within the chamber is a trapezoidal shape constructed from three black Plexiglas walls, which open to the touchscreen (Dimensions: 20 cm x 18 cm screen-reward tray x 24 cm at screen, or 6 cm at reward tray). This shape is specially designed to direct the attention of the animal towards the touchscreen and reward tray (or food magazine). Located centrally either on the rear aluminum wall or attached to the wall opposite the touchscreen (depending on the test, see specific description of behavioral procedures below) is a food magazine linked to a liquid dispenser pump (Strawberry milkshake (Quebon) serves as food reward). A light emitting diode illuminates the food magazine. Computer graphic white square stimuli are presented on the touchscreen. Depending on the task, different black aluminum masks are attached to the face of the screen approximately 1.5 cm from the surface of the display. These masks serve to restrict the mouse’s access to the display except through response apertures. A miniature infrared camera is installed above the chamber to allow monitoring of the animals’ behaviour. Animal activity is recorded via infrared photobeams traversing the sidewalls of the chamber at the front (screen) and rear (reward tray). The apparatus and online data collection for each chamber are controlled by a Dell computer connected to an Animal Behavior Environmental Test system (Lafayette Instruments) using the Whisker control system for research ^6^. All experiments were performed with the houselight off.

Operant Self-Administration of chocolate flavor pellets

Mice were tested in operant chambers (Med Associates) and nosepoke responses were recorded by MedPC IV software. Mice were trained to obtain a chocolate pellet (Dustless Precision Pellets®, 20 mg, Rodent Purified Diet, Chocolate Flavor, Cedarlane Laboratories) by means of an operant response associating the food pellet delivery to the right hand hole. Both the active and inactive holes were lit, by nose poking the inactive hole the animal received no pellet but no punishment. The beginning of each operant responding session was signaled by turning on a house light placed on the ceiling of the box and a fan to provide ventilation and white noise. Animals were trained under a fixed-ratio (FR) 1 schedule of reinforcement in 20 minute daily sessions. The criteria for acquisition of operant responding were achieved when mice maintained a stable responding with <20% deviation from the mean of the total number of food pellets earned in three consecutive sessions and a minimum of 10 reinforcers per session. The animals that reach this criterion were then moved onto an FR3 schedule. Delay to reach criterion during the last session under FR1 and FR3 were recorded. Only animals that reach a criterion were included in the latency to reach criterion analysis (10 rewards under FR1; 10 or 25 rewards under FR3). At the end of the FR3 procedure, the same animals were tested in 5-min extinction session conducted in the absence of food reinforcement.

**References**

1 Matthes, H. W. *et al.* Loss of morphine-induced analgesia, reward effect and withdrawal symptoms in mice lacking the mu-opioid-receptor gene. *Nature* **383**, 819-823, doi:10.1038/383819a0 (1996).

2 Mechling, A. E. *et al.* Deletion of the mu opioid receptor gene in mice reshapes the reward-aversion connectome. *Proc Natl Acad Sci U S A* **113**, 11603-11608, doi:10.1073/pnas.1601640113 (2016).

3 Chakravarty, M. M. *et al.* Performing label-fusion-based segmentation using multiple automatically generated templates. *Hum Brain Mapp* **34**, 2635-2654, doi:10.1002/hbm.22092 (2013).

4 Arefin, T. M. *et al.* Remodeling of Sensorimotor Brain Connectivity in Gpr88-Deficient Mice. *Brain Connect* **7**, 526-540, doi:10.1089/brain.2017.0486 (2017).

5 Nichols, T. & Hayasaka, S. Controlling the familywise error rate in functional neuroimaging: a comparative review. *Stat Methods Med Res* **12**, 419-446, doi:10.1191/0962280203sm341ra (2003).

6 Cardinal, R. N. & Aitken, M. R. Whisker: a client-server high-performance multimedia research control system. *Behavior research methods* **42**, 1059-1071, doi:10.3758/brm.42.4.1059 (2010).

7 Horner, A. E. *et al.* The touchscreen operant platform for testing learning and memory in rats and mice. *Nature protocols* **8**, 1961-1984, doi:10.1038/nprot.2013.122 (2013).
